# Association study between DNA methylation and genetic variation of *APOE* gene with the risk of coronary artery disease

**DOI:** 10.22099/mbrc.2018.30955.1352

**Published:** 2018-12

**Authors:** Habib Ghaznavi, Ali Asghar Kiani, Mohammad Soleiman Soltanpour

**Affiliations:** 1 Department of Pharmacology, School of Medicine, Zahedan University of Medical Sciences, Zahedan, Iran; 2 Department of Hematology and Blood Transfusion, School of Allied Medical Sciences, Lorestan University of Medical Sciences, Khorramabad, Iran; 3 Department of Medical Laboratory Sciences, School of Paramedical Sciences, Zanjan University of Medical Sciences, Zanjan, Iran

**Keywords:** Coronary artery disease, Polymorphism, Methylation, Apo-lipoprotein E

## Abstract

Coronary artery disease (CAD) is a common health problem with a high rate of disability and death. Dyslipidemia and altered metabolism of Apo-lipoproteins are involved in the CAD pathogenesis. The current study investigated two common polymorphisms (rs429358 and rs7412) and promoter DNA methylation status of *APOE* in the Iranian CAD patients and control subjects. Two hundred angiographically documented CAD patients and 200 control subjects were included in the study. The *APOE* polymorphism analysis was done by PCR-RFLP technique and DNA methylation status was evaluated by methylation specific PCR. The assay of lipid levels was conducted using standard colorimetric protocols. Results indicated that the frequency of ε3/ε4 and ε2/ε3 genotypes was significantly more common in CAD group compared with control group. Relative to wild type genotype (ε3/ε3), CAD patients with ε3/ε4 and ε2/ε3 genotypes displayed significantly higher concentration of total-cholesterol and LDL-cholesterol. The frequency of DNA methylation of *APOE* was similar between the two studied groups. However, the methylation frequency of *APOE* gene was significantly higher in triple stenotic vessels relative to single stenotic vessels (P=0.032). In conclusion The present study indicated that the rs429358 and rs7412 polymorphisms are significantly risk factors for development and severity of CAD. Also, *APOE* methylation status may be involved in the severity but not in the development of CAD.

## INTRODUCTION

Coronary artery disease (CAD) is defined as a multicausal disease with a detrimental health and economic consequences. Several risk factors including environmental, genetic and epigenetic has been implicated in the development of CAD [1]. Dyslipidemia as a significant risk factor has a major influence on the development of CAD [2]. In addition to acquired factors, dyslipidemia may be caused by genetic and epigenetic alterations in some genes involved in the lipid metabolism [3-5]. 

Apolipoprotein E (APOE) is a 299 amino acids containing glycoprotein involved in transportation and metabolism of lipids. APOE is expressed on a wide variety of lipoprotein particles [6, 7]. It plays pivotal roles in the clearance of various plasma lipids by acting as a ligand for appropriate hepatic receptors [6]. Two common single nucleotide polymorphisms, namely rs429358 and rs7412, produces three alleles, ε2, ε3 and ε4 that co-dominantly create six different genotypes (ε2/ε4, ε2/ε3, ε3/ε4, ε3/ε3,ε2/ε2, ε4/ε4) [8, 9]. Three major protein isoforms (E2, E3, E4) are encoded by these different alleles of *APOE* gene [10]. The E3 is the wild type isoform which acts as a scavenger for a variety of metabolized lipoproteins from the circulation[11, 12]. Whereas, the E2 and E4 isoforms have altered affinity for LDLR that could affect lipid profile and the development of CAD [11, 12]. APOE isoforms causes a variability ranges of 2–16% in the LDL cholesterol levels [13, 14]. The association of APOE polymorphisms with the risk of CAD has been assessed in numerous studies with inconsistent findings [3, 15]. Abnormal DNA methylation in pivotal genes associated with lipid metabolism may play a significant role in the development of CAD [16]. DNA methylation is a key mechanism involved in the regulation of gene transcription activity [17]. *APOE* hyper-methylation has been identified in several age related pathologies including impaired cognitive function, alzheimeŕs disease and schizophrenia [18-20]. However, information regarding the possible involvement of *APOE* hyper-methylation in the development and severity of CAD is scarce. The current study was aimed to compare the DNA methylation status of *APOE* promoter and the frequency of *APOE* polymorphisms in a group of CAD patients and control group.

## MATERIALS AND METHODS

**Study population: **The population under study consisted of 200 CAD patients referred to angiography center of Moussavi education and treatment center of Zanjan and 200 matched controls. Angiography technique was used for diagnosis of CAD. The patients were classified into three groups including single, double, and triple vessel stenosis patients based on the presence of ≥50% stenosis in the vessels. The main inclusion criteria for CAD group consisted of the luminal stenosis of ≥50% in at least one major coronary vessel. The exclusion criteria for CAD patients were the presence of infectious disease, auto immune disorders, inflammatory disease, cancer and use of lipid-controlling drugs. The selection of control subjects were based on careful examination of a cardiovascular specialist. The exclusion criteria for control subjects were the presence of any acute and chronic disease, febrile and inflammatory disease or family history of CAD. All study participants completed a questioner regarding the presence of family history of heart disease, hypertension, diabetes, smoking habits, dyslipidemia and other inflammatory conditions. The consent form was signed by all of the participants in the study. The approving of the study was conducted by ethical committee of Zahedan University of Medical Sciences (Ethical code: IR.ZAUMS. REC.1396.5), Zahedan, Iran**.**

**Biochemical assays: **Following a 12 hours overnight fasting, 5 ml blood was collected in EDTA anti-coagulated tubes and centrifuged immediately. The plasma fraction was separated and used for biochemical assays. The biochemical assays of each sample including glucose and lipid profile was conducted with calorimetric methods using a Mindray auto-analyzer (BS-200) according to instruction of assay kits (Pars Azmoon Co, Tehran, Iran).

**DNA isolation and bisulphite treatment: **DNA was extracted from whole blood leukocytes using the YTA Blood DNA Kit (Yekta tajhiz Azma, Iran), according to instructions prepared by manufacturer. The quality and quantity of purified DNA was assessed by a Nanodrop spectrophotometer. The EpiTect Bisulfite Kit (Qiagen, Germany) was used to treat approximately 2 µg of extracted DNA by sodium bisulphite during which the unmethylated cytosines converted into the corresponding uracils, while the methylated cytosines remained unchanged in their positions. The converted DNA was stored at -20 ºC until analysis.

**Methylation specific PCR: **The Methylation sensitive PCR (MSP) was used to assess the methylation status of *APOE* gene in 100 randomly selected CAD patients (51 males, 49 females) with the mean ages of 61.3±12.8 and 100 randomly selected control subjects (52 males, 48 females) with the mean ages of 58.9±10.3. The primers specific for methylated and un-methylated status of APOE promoter were designed using a special website [22]. The sequence of primers for un-methylated gene were F:5'GGT TGG GGT TAG TTG ATG TTT ATT AT3', R:5'AA AAA AAC TAA ACT CCT AAT TCA AA3' and for methylated gene were F:5'GTT GGG GTT AGT TGA TGT TTA TTA C3', R:5AAA AAA ACT AAA CTC CTA ATT CGA A3'. For amplification of target sequence, MSP analysis was done in a final volume of 20 µl comprising 10µl hot start master mix (EpiTect MSP Kit, Qiagen, Germany), 0.5 µM of each primer and 2µl of bisulphite converted DNA. Appropriate controls (EpiTect PCR Control DNA Set, Qiagen, Germany) were used in each set of PCR reaction. The annealing temperature for methylated and un-methylated PCR reactions were 57ºC and 58ºC, respectively. Following the completion of MSP reaction, the electrophoresis of amplified products on a 3% agarose gel revealed a 203 and 204bp for methylated and un-methylated state, respectively. 

**Polymorphism analysis: **Genotypes for rs429358 and rs7412 polymorphisms were determined using PCR-RFLP. The polymorphic site of APOE was amplified using the following primer sets, F:5'TCC AAG GAG CTG CAG GCG GCG CA3'; R: 5'GCC CCG GCC TGG TAC ACT GCC A3'. The digestion of amplified fragment (218bp) was conducted in a 20µl reaction volume containing 5U *Hae*II and 5U *Afl*III restriction enzyme, 10µl PCR products, 2µl 10x buffer and appropriate volume of water. The digested product was electrophoresed on a 4% agarose gel. The APOE alleles were appeared at 168, 145 and 195 bp for E2, E3 and E4 isoforms, respectively. 

**Statistical analysis: **The numerical values as presented mean ± SD were compared using Student *t*-test or ANOVA test. Chi square test or Fisher's exact tests was performed to assess the differences between categorical variables. Also, in order to evaluate the independent effect of each risk factor on the development of CAD, binary logistic regression analysis was done. Results were analyzed using statistical software package SPSS version 16 (SPSS Inc., Chicago, Ill) and a P value less than 0.05 was considered statistically significant.

## RESULTS

The demographic, clinical and laboratory characteristics of the study population are presented in Table 1. Statistical analysis indicated no significant difference in the sex distribution (P=0.84) and mean age differences (P=0.67) between the two groups.

**Table 1:** The demographic, clinical and biochemical characteristics of study population

**Parameters**	**CAD group n=200**	**Control group n=200**
Age	60.2 ± 12.4	59.1 ± 10.6
Sex (M/F)	103/97	105/95
TG	194.1 ± 84.5	173.4 ± 73.9
TC	203.8 ± 60.6	168.1 ± 47.8
LDL-C	101.2 ± 35.5	91.1 ± 33.3
HDL-C	39.1 ± 9.3	46.5 ± 13.5
Hypertension	46 (23%)	19 (9.5%)
Diabetes	50 (25%)	22 (11%)
Smoking	52 (26%)	21 (10.5%)

TG: triglyceride; TC: total cholesterol; LDL-C^: ^low density lipoprotein cholesterol; HDL: high density lipoprotein cholesterol.

Table 2 represents the genotype frequency and allele distribution of two APOE polymorphisms. The frequency of ε3/ε4 and ε2/ε3 genotypes was significantly higher in CAD group than control group. The ε2 allele increased the risk of CAD by 2.39 fold (P=0.016) while the ε4 allele increased the CAD risk by 2.15 fold (P<0.001).

**Table 2:** The genotypic and allelic distribution of *APOE* polymorphisms in the study groups

**Genotype/Allele**	**CAD group**	**Control group**	**OR (95% CI)**	**P**
ε3/ε3	110 (55%)	154 (77%)	Ref	-
ε2/ε3	22 (11%)	10 (5.0%)	3.08 (1.40-6.76)	0.004
ε3/ε4	62 (31%)	32 (16.0%)	2.71 (1.65-4.43)	<0.001
ε2/ε4	3 (1.5%)	2 (1.0%)	2.1 (0.34-12.78)	0.65
ε4/ε4	3 (1.5%)	2 (1.0%)	2.1 (0.34-12.78)	0.65
ε3	304 (76%)	350 (87.5%)	Ref	-
ε2	25 (6.25%)	12 (3%)	2.39 (1.18-4.85)	0.016
ε4	71 (17.75%)	38 (9.5%)	2.15 (1.40-3.28)	<0.001

Statistical analysis using ANOVA test indicated that CAD patients and control subjects with ε3/ε4 and ε2/ε3 genotype had significantly higher levels of TC and LDL-C relative to wild type genotype (ε3/ε3) (Table 3). Also, the comparison of *APOE* genotypes between different categories of CAD patients indicated that the prevalence of ε3/ε4 genotype (in comparison to ε3/ε3 genotype as reference group) was significantly more common among CAD patients with three stenotic vessels (30/28) relative to CAD patients with one stenotic vessels (15/47) (OR=3.35, 95% CI =1.54-7.30; P=0.002). Next, we investigated the frequency of *APOE* DNA methylation in the study population. Statistical analysis using Chi-square test indicated no significant difference between the two groups (54 out of 100 CAD patients *vs*. 43 out of 100 controls, OR=1.25; 95% CI=0.77-2.04; P=0.359). However, the frequency of methylated APOE gene was significantly more common among CAD patients with three SV relative to CAD patients with one SV (24 out of 34 *vs*.16 out of 36, OR=3.01; 95% CI=1.11-8.06; P=0.032).

**Table 3:** lipid profile distribution across different genotypes of *APOE* polymorphisms

**Genotype**	**CAD group**				**Control group**			
	**ε3/ε3**	**ε2/ε3**	**ε3/ε4**	**P**	**ε3/ε3**	**ε2/ε3**	**ε3/ε4**	**P**
TG^a^	181.1±87.7	192.4±98.3	219.5±66.9	0.015	173.5±71.6	172.9±92.9	168.3±83.0	0.887
TC^a^	192.8±59.5	215.1± 64	219.7±58.4	0.013	162.4±48.8	173.9±51.9	194.3±30.9	0.002
LDL-C^a^	93.4±33.7	103.8±46.3	114.2±29.8	0.001	86.9±32.7	101.1±37.2	107.2±29.7	0.040
HDL-C^a^	39.2±9.2	37.9±11.5	39.2±8.8	0.829	46.2±13.2	49.9±15.8	47.1±14.9	0.685

a The values are presented as mg/dL. For abbreviations see Table 1.

Finally, we performed a binary logistic regression analysis to assess the independent effect of each covariate on the risk of CAD development. Results indicated that the ε3/ε4 genotype, smoking, diabetes, hypertension, TC, LDL-C, and HDL-C as significant and independent risk factors for CAD development. All of these covariates increased the risk of CAD except for HDL-C which was associated with a reduced risk of CAD (Table 4). 

**Table 4:** Binary Logistic regression analysis of association between each variable and risk of CAD

**Variable**	**Crud values**		**Adjusted values**	
	**OR (95% CI)**	**P-value**	**OR (95% CI)**	**P-value**
Hypertension	2.16 (1.22- 4.83)	0.002	2.14 (1.34- 4.74)	0.017
Diabetes	2.71 (1.87-7.86)	0.005	2.68 (2.15-7.23)	0.021
Smoking	3.83 (1.23-7.12)	<0.001	3.43 (1.42-6.23)	0.003
TG	1.01 (1.00-1.01)	0.011	1.00 (1.00-1.01)	0.061
TC	1.01 (0.96-1.04)	<0.001	1.00 (0.98-1.02)	<0.001
HDL-C	0.93 (0.91-0.96)	0.002	0.94 (0.90-0.98)	0.007
LDL-C	1.01 (0.96-1.02)	0.005	1.00 (0.98-1.01)	0.011
ε3/ε4	2.71 (1.65-4.43)	<0.001	2.28 (1.12-3.64)	0.002

For abbreviations see Table 1.

## DISCUSSION

CAD is a common and multifactorial disease with a high rate of disability and mortality. Dyslipidemia and alteration in apo-lipoprotein metabolism is a common finding in CAD patients [19]. The present study investigated the prevalence of some common polymorphisms and methylation frequency of *APOE* in an Iranian sub-population of CAD patients. Present results indicated the significantly high prevalence of ε2 and ε4 allele in CAD group relative to control group. The frequency of ε4 allele in the present study (17.75%) was comparable with the reported frequency in Dutch population (16.3%) [22] and was higher than that of Italian (9.1%) [22], Egyptian (9.4%) [11] and Turkish (8%) population [5]. However, it was lower than the reported frequency in France (22.9%) [3] and African American (27.7%) population [23]. Results of the current study identified Apo ε2 allele as a risk marker for CAD, while some other studies reported this allele as a risk protective factor [23, 24]. 

Also, our results showed that the presence of ε3/4 genotype increased the risk of CAD. This result is in agreement with some previous studies [11, 15, 25, 26] and in conflict with some others [3, 5]. These conflicting findings of association studies may be related to numerous factors including differences in study design, sample size, sample selection criteria, different racial and genetic background of studied populations and gene- gene and gene-environmental interactions [27]. The ε4 allele may affect the development and severity of CAD indirectly via influencing the lipid levels, as our results indicated higher levels of LDL-C and TC in carriers of e4 allele. Similarly, various studies have reported an atherogenic lipid profile in ε4 allele carriers [3, 11, 28]. It should be noted that impaired recycling of ApoE4 between triglyceride rich lipoprotein remnants could lead to decreased cholesterol efflux and elevation in cholesterol levels [4]. The current study identified that carriers of ε3/4 genotype had higher number of stenotic vessels in relative to carriers of ε3/3 genotype of *APOE* which confirmed the function of this genetic variant in increasing the severity of disease. Similarly, Li et al., in a recently published study indicated that the number of stenotic vessels was significantly higher in carriers of e3/3 genotype relative to carriers of ApoE4. [24] The antioxidant activities of ApoE isoforms were as follows: ApoE2>ApoE3>ApoE4. The association of ε4 allele with the severity of CAD may be explained by the low antioxidant activity of ε4 allele that causes oxidized LDL-C which subsequently triggers foam cell formation and atherosclerosis [29, 30]. 

Several previous studies have suggested that epigenetic modifications may contribute to the development of CAD [1, 31, 32]. Although, in the present study, a high frequency of Apo-E DNA methylation was observed in CAD group than that of control group, no statistically significant difference was seen (P>0.05). The association between *APOE* DNA methylation and prevalence of some age related disease has been studied in numerous studies [18-20]. Liu et al., reported an inverse correlation between methylation at multiple CpGs sites in *APOE* with delayed recall during normal cognitive aging [18]. The recent study by Kordi-Tamandani et al., reported no significant association between the *APOE* gene methylation and development of schizophrenia [19]. More recently, in agreement with the present study, Karlsson et al., indicated that higher DNA methylation levels in the promoter region of *APOE* did not influence the risk of coronary artery disease but increased the risk of dementia and Alzimer disease [20]. Although, we did not find any significant differences in the frequency of *APOE* DNA methylation between CAD group and control group, comparison of CAD patients with one, two and three or more stenotic vessels revealed high frequency of DNA methylation in CAD patients with three stenotic vessels. This result may confer a role for Apo-E DNA methylation in determining the severity of CAD. However, more complementary studies with large sample size are required to confirm this preliminary finding. Some limitation exists in our study including (I) the gene expression levels of Apo-E gene was not investigated (II) the plasma levels of Apo-E and other apo-lipoproteines was not determined.
